# Factors Affecting Health-Related Quality of Life in Multimorbidity

**DOI:** 10.3390/healthcare9030334

**Published:** 2021-03-16

**Authors:** Eunmi Lee, Sunkyung Cha, Geun Myun Kim

**Affiliations:** 1 Department of Nursing, Hoseo University, Asan 31499, Korea; sweetbear2@hanmail.net; 2 Department of Nursing Science, Sun Moon University, Asan 31460, Korea; 3 Department of Nursing, Gangneung-Wonju National University, Wonju 26403, Korea; gmkim@gwnu.ac.kr

**Keywords:** multimorbidity, Andersen’s model, health-related quality of life, chronic disease management

## Abstract

We investigated the effect of predisposing, enabling, need factors, and health behaviors on health-related quality of life (HRQoL) of patients with multimorbidity according to Andersen’s model. This study is a secondary analysis of population-based cross-sectional surveys. Data from 328 patients with multimorbidity (≥3 co-occurring chronic diseases) from the 6th/7th Korea National Health and Nutrition Examination Surveys were analyzed using logistic regression. Patients ≥65 years, without private insurance, with poor subjective health, unmet medical needs, and/or limited activity were more likely to experience mobility problems. Self-care problems were more likely among those without private insurance and/or with limited activity. Patients lacking living security, with poor subjective health, limited activity, and/or who smoked were more likely to experience problems performing usual activities. Pain/discomfort was more likely among females, Medicaid beneficiaries, and patients with limited activity and/or with poor subjective health. Patients with poor subjective health, limited activity, and/or unmet medical needs were more likely to experience anxiety/depression. The investigation of HRQoL in multimorbidity should consider predisposing, enabling, need factors, and health behaviors. Interventions addressing movement restrictions and personalized care based on HRQoL domains should be prioritized.

## 1. Introduction

### 1.1. Background

As medical technology advances and human life expectancy increases, the prevalence of chronic diseases has raised as a result of population aging. Chronic diseases are a leading cause of disability and death worldwide, accounting for 60% of global deaths [1]. However, the management of health-related habits such as smoking, drinking alcohol, physical activity, and diet, which contribute to chronic diseases, has not improved. The prevalence of multimorbidity is also on the rise, and the OECD recently identified chronic disease as a critical issue faced by many countries [2]. Multimorbidity refers to two or more co-occurring diseases [3,4,5] and is prevalent in 23% of the general population, affecting 65% of individuals aged ≥65 years, and 55% of patients with chronic diseases [6].

In South Korea, the average patient with chronic disease aged ≥50 years has two or more diseases. On average, patients aged ≥60 years have 3, and patients aged ≥70 years have 3.5 conditions [7]. Multimorbidity is expected to increase with population aging [8], thus requiring the implementation of health-related policies and continuous management of co-occurring diseases, with attendant rising treatment frequency and medical costs [9]. In South Korea, the focus of multimorbidity studies has been on their prevalence, and a few studies have focused on chronic disease combinations and the cost of medical treatments related to multimorbidity [8,10].

Patients with multimorbidity must receive continuous treatment and maintain their quality of life (QoL). The QoL of older adults is especially important when considering the prevalence of multimorbidity in isolation; however, research using samples of younger adults is also needed, since there may be a higher prevalence of multimorbidity among adults aged <65 years depending on the population distribution ratio in a given population group [8,11]. Therefore, there is a need to examine QoL in all adults with multimorbidity.

Health-related QoL (HRQoL) is a subjective, multidimensional concept expressing an individual’s satisfaction with functional abilities related to his/her health [12]. It is useful for determining individuals’ functional abilities and wellness [13], and as an index for policies in the public health sector when determining and improving community residents’ health status. Most research on the factors influencing HRQoL has focused on individual characteristics, such as financial status, education level, occupation, emotional state (e.g., depression, anxiety, stress), and physical health, in vulnerable elderly populations such as those living alone or receiving a basic income [14,15,16,17]. Thus, applying the results of these studies to patients with multimorbidity has limitations. Although research has reported that HRQoL is affected by the interaction of individuals’ various characteristics, including emotional state and socio-environmental factors, research comprehensively examining the physical, mental, and social aspects of QoL in patients with multimorbidity has not been conducted in South Korea.

Andersen’s model was developed in the 1990s to analyze and explain health outcomes such as customer satisfaction or QoL in relation to health services [18], and suggests that predisposing, enabling, and needs factors influence individuals’ health outcomes. In Korea, Andersen’s model has usually been used in QoL research with older adults [19,20,21]. Additionally, the model was designed to explore how and why individuals and families utilize general healthcare, welfare services in socially structured environments, and the effect on outcomes, including health status and health satisfaction. The model is multidimensional and includes contextual and individual characteristics as well as health behaviors and outcomes [22].

According to Andersen’s model, the primary factors determining health behaviors are socio-demographic characteristics, public health systems, and external environmental factors, which also affect health outcomes such as consumer satisfaction and QoL [23,24]. Studies conducted in Korea based on this model include those examining older adults’ ability to perform usual daily activities and use medical services, and their oral HRQoL [21,24,25]. In a systematic review about health service use by Babitsch et al. (2012), age, marital status, gender, education level, and race were identified as predisposing factors; household income, financial condition, health insurance availability, and usable resources were enabling factors; and health condition, subjective health status, and other various diseases were need factors. This study aimed to identify factors influencing each dimension of HRQoL in patients with multimorbidity in Korea using Andersen’s model. In the future, this information could be used as a basis for establishing health and welfare policies related to multimorbidity.

### 1.2. Purpose

In this study, we investigated the effect individual characteristics as primary factors (i.e., predisposing, enabling, and need factors) and health activities as secondary factors on HRQoL in patients with multimorbidity based on the application of Andersen’s model.

## 2. Methods

### 2.1. Design

A cross-sectional survey was used to identify factors affecting the HRQoL of patients with multimorbidity (i.e., three or more co-occurring chronic diseases) based on Andersen’s model.

### 2.2. Participants

We analyzed data collected in 2014 and 2015 in the 6th Korea National Health and Nutrition Examination Survey (KNHNES) (2013–2015) and in 2016 in the 7th KNHNES (2016–2018). These surveys assess health status, health behavior, food consumption, and nutritional status of all Korean nationals over the age of 1 year. The KNHNES is a single-year survey conducted over 3 years. It was initiated in 1998 and has been used as an annual rolling sample survey method since 2007 to help establish public health policies, involving setting goals for and evaluating the comprehensive national health improvement plan, and developing other health improvement plans [26]. In this study, data from the most recent 3 years (2014, 2015, and 2016) were used.

**Figure 1:**
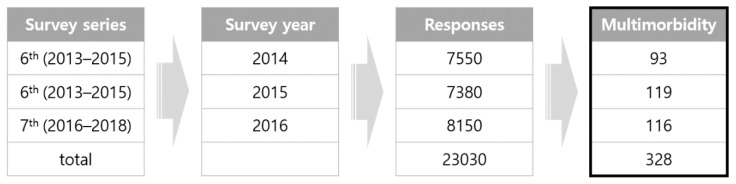
Respondents of patient with multimorbidity (*N* = 328) (South Korea, 2014–2016).

Among the 27 chronic diseases studied in the KNHNES, respondents with at least 3 of the 13 chronic diseases defined by the World Health Organization (WHO) (i.e., hypertension, stroke, cardiac infarction, angina, diabetes, and stomach, liver, colon, breast, cervical, lung, thyroid, and other cancers) occurring concurrently were defined as having multimorbidity. In 2014, the second year of the 6th survey (2013–2015), 7550 responses were recorded; 7380 responses were recorded in 2015, the third year of the 6th survey; and 8150 responses were recorded in 2016, the first year of the 7th survey (2016–2018). The number of patients with at least three chronic diseases [5] was 93, 119, and 116 in 2014, 2015, and 2016, respectively. The total number of respondents whose data were used in this study was thus 328 (Figure 1). 

### 2.3. Measurements

HRQoL (dependent variable), was examined using the EQ-5D-3L after the KCDC obtained permission from the EuroQol Group, and comprised five dimensions: mobility, self-care, usual activities, pain/discomfort, and anxiety/depression (emotional state: e.g., depression, anxiety, stress). Items are rated on a 3-point scale (1 = “no problem”, 2 = “some problem”, and 3 = “extreme problem”). In this study, each HRQoL domain was assessed as a dichotomous variable: “no problem” and “problem”.

In the Andersen model, contextual characteristics and individual characteristics are factors influencing the use of medical services along with health conditions, which are defined as predisposing factors, enabling factors, and need factors. Predisposing factors are inherent socio-demographic characteristics, such as gender, age, education level, and marital status. Enabling factors are those facilitating or inhibiting the use of medical service or health conditions, include the receipt of basic living security, health insurance type, existence of private insurance, household income, living with family, and limited activity. Need factors are elements that require medical services such as the presence of a disease and include the number of chronic diseases, subjective health, and the presence of unmet medical needs. Lastly, health behaviors are health-related activities, such as smoking, drinking alcohol, physical activity, and health screenings.

The model factors were categorized into predisposing, enabling, and need factors, and health behaviors. Gender, age (<65, ≥65), education level (≤high school graduate, >high school graduate), and marital status (presence or absence of spouse) were considered as predisposing factors. Receipt of basic living security, insurance type (national health insurance, Medicaid), private insurance household income (in 10,000 South Korean won), living with family, and limited activity were considered enabling factors. The number of chronic diseases, subjective health status (good or poor), and having unmet medical needs were considered as need factors. Lastly, smoking, drinking, physical activity, and health screenings were considered as health behaviors.

### 2.4. Statistical Analysis

SPSS 20.0 was used to analyze the data. Frequency analysis and descriptive statistics were performed for all variables as general characteristics. Logistic regression analysis was conducted to identify the factors affecting each HRQoL dimension (dependent variables); Andersen’s model variables were added in the following order: predisposing factors, enabling factors, need factors, and health behaviors.

## 3. Results

### 3.1. General Characteristics of Multimorbidity

Participants’ general characteristics are shown in Table 1. Regarding predisposing factors, 164 (50%) respondents were male; 240 (73.2%) were ≥65 years (26.8%); 284 (87.6%) were ≤high school graduate, while 40 (12.45%) were >high school graduate; 225 (68.6%) had a spouse.

Regarding enabling factors, 277 (84.5%) respondents had never received basic living security. Most respondents (281, 87.85%) had national health insurance, and 39 (12.2%) had Medicaid; moreover, 117 (35.7%) had private insurance, and 211 (64.3%) did not have any. The average household income was 2,101,000 won per month; 257 (78.3%) respondents were living with family; 89 (27.1%) respondents displayed limited activity.

Regarding need factors, 275 (83.9%) respondents had three chronic diseases, while 46 (14.0%) had four, and 7 (2.15%) had five (mean number of diseases = 3.2); 205 (62.5%) respondents had poor subjective health status; and 41 (12.5%) respondents had unmet medical needs.

Regarding health behaviors, 59 (18.4%) respondents smoked; 153 (47.7%) drank alcohol; 42 (12.9%) respondents exercised; and 191 (58.4%) underwent health screenings.

Regarding the HRQoL domains, 174 (53.2%) respondents had mobility problems, 72 (22.0%) had self-care problems, 141 (43.0%) had problems with usual activities, 152 (46.3%) had pain/discomfort, and 88 (26.8%) had anxiety/depression.

### 3.2. Factors Affecting HRQoL

In terms of factors affecting the mobility dimension of HRQoL (Table 2), respondents ≥65 years, without high school education, and/or without a spouse were more likely to have problems with mobility in Model 1 (predisposing factors). Respondents aged ≥65 years, without high school education, without private insurance, and/or with limited activity were more likely to have problems with mobility in Model 2 (predisposing + enabling factors). Respondents aged ≥65 years, without high school education, without private insurance, with limited activity, who had poor subjective health status, and/or who had unmet medical needs were more likely to have problems with mobility in Model 3 (predisposing + enabling + need factors). Respondents ≥65 years, without private insurance, with limited activity, who had poor subjective health status, and/or who had unmet medical needs were more likely to have problems with mobility in Model 4 (predisposing factors + enabling factors + need factors + health behaviors).

In terms of factors affecting the self-care dimension of HRQoL (Table 3), Model 2–4, respondents without private insurance and/or who had limited activity were more likely to have problems with self-care, while other factors were not significant.

Regarding factors affecting the usual activities dimension of HRQoL (Table 4), Respondents who received Medicaid, without private insurance, and/or with limited activity were more likely to have problems with usual activities in Model 2. In Model 3, respondents who had never received basic living security, who had Medicaid, who had limited activity, and/or who had poor subjective health status were more likely to have problems with usual activities. In Model 4, respondents who had never received basic living security, with limited activity, who had poor subjective health status, and/or who smoked were more likely to have problems with usual activities.

In terms of factors affecting pain/discomfort dimension of HRQoL (Table 5), females were more likely to experience problems than males in Model 1. Respondents who were female, who received Medicaid, and/or who had limited activity were more likely to experience pain/discomfort in Model 2. In Models 3 and 4, respondents who were female, who received Medicaid, who had limited activity, and/or who had poor subjective health status were more likely to experience pain/discomfort.

In terms of factors affecting the anxiety/depression dimension of HRQoL (Table 6), respondents who were <65 years and/or had limited activity were more likely to experience anxiety/depression in Model 2. In both Models 3 and 4, respondents with limited activity, who had poor subjective health status, and/or who had unmet medical needs were more likely to experience anxiety/depression.

## 4. Discussion

The public health sector in Korea is currently facing rapid population aging with the consequent increase in prevalent multimorbidity and related medical expenses. Despite the long average life expectancy in Korea, chronic diseases contribute to a relatively low healthy life expectancy; thus, increasing the QoL of patients with multimorbidity may be an important policy issue. Previous studies on multimorbidity concentrated on overall HRQoL (EQ-5D index) [27], whereas recent studies have placed more importance on examining each dimension [28,29]. As QoL is a complex concept encompassing all elements for a satisfactory life [30], it is necessary to develop specific strategies to improve each dimension of QoL by inspecting the factors that influence it. In our study, Model 4, which included all of the predisposing, enabling, need factors, and health behaviors, had the greatest influence on all five dimensions. This indicates that all four factor types must be considered when examining HRQoL.

Regarding the specific HRQoL dimensions, in Models 1–4, respondents ≥65 years, without private insurance, with limited activity, with poor subjective health status, and/or with unmet medical needs were more likely to have problems with mobility. Our results support those from previous studies suggesting that physical function declines with age, thereby reducing QoL in elderly persons [31]. Having unmet medical needs, which refers to cases where treatment/examination is needed but not received, was attributable to time limitations as well as poor access to healthcare institutions.

As for self-care, no factors had statistical significance in Model 1, whereas in Models 2–4, respondents without private insurance (63.5%) and/or with limited activity were more likely to have problems. This implies that most of these patients did not have a secure financial status, which is supported by the fact that 49.1% of the respondents were at the bottom end of the income distribution. Those with limited activity were more likely to have problems with self-care, which can be related to poor financial conditions, since limited activity is related to low income [32].

Regarding usual activity, no factors had statistical significance in Model 1, whereas in Models 2–4, respondents who had never received basic living security, who received Medicaid, and/or without private insurance were more likely to have problems, which suggests that poor financial status, indicated by Medicaid and lack of private insurance, leads to decreased ability to perform usual activities. Although a social guarantee system such as basic living security is limited, it can still improve basic QoL. This implies that a policy that includes individuals who are not covered by such social systems or private insurance needs to be implemented. In Model 4, respondents who had never received basic living security, with limited activity, who had poor subjective health status, and/or who smoked were more likely to have problems with usual activities. As an individual habitual health-related behavior, smoking affected daily activities (work, studying, housework, and leisure) across all five dimensions of the HRQoL. Smoking must be strictly controlled, as it is one of the major causes of chronic disease, ultimately leading to multimorbidity. A special smoking cessation program for patients with multimorbidity together with an efficient stress management program may be helpful for patients with multimorbidity who have difficulties with smoking cessation. Moreover, subjective health status involves a comprehensive awareness of one’s health and the social environment surrounding it; therefore, establishing social systems that improve these subjective health conditions is necessary to increase daily activity in patients with multimorbidity.

Regarding pain/discomfort, gender was a significant factor in Model 1, and gender, type of insurance, and limited activity were significant in Model 2. In Models 3 and 4, respondents who were female, who received Medicaid, with limited activity, and/or with poor subjective health status were more likely to experience pain/discomfort. This is in line with the results of previous studies reporting that females are more likely to experience pain related to musculoskeletal, circulatory, respiratory, and digestive systems [33]. A management system for patients with multimorbidity must be established considering differences in pain/discomfort, and gender is an important factor to take into account, since studies conducted in other countries using the EQ-5D also showed that females were more likely to experience difficulties in this domain.

According to studies on multimorbidity and QoL, multimorbidity has an impact on the physical dimensions of QoL, but little is known about the effect on social and emotional dimensions of QoL [34]; thus, assessing the anxiety/depression domain is especially important for patients with multimorbidity. No factors were significant in Model 1, whereas in Model 2, age and limited activity were significant. In Models 3 and 4, respondents with limited activity, with poor subjective health status, and/or with unmet medical needs were more likely to experience anxiety/depression. According to a study on patients with chronic diseases by the Korea Health Panel [32], vulnerable social groups (older persons, those with low income, persons with disabilities, and those with a low educational level) had a higher level of limited activity and unmet medical needs; moreover, patients with chronic diseases who had limited activity were more likely to have unmet medical needs than those without limited activity. The percentage of respondents with multimorbidity and unmet medical needs in the present study was 12.5%, which is lower than 23.7% among young adults and 21.7% among older adults reported by the Korea Health Panel [27]. Additionally, our participants had an average of 3.2 chronic diseases, and no participant had more than 6 chronic diseases, whereas the study conducted by Korea Health Panel reported a mean of 5.75 diseases, and 31.2% of the study participants had at least 7 chronic diseases. Considering such results and the influence of limited activity and unmet medical needs on anxiety/depression in patients with multimorbidity, adequate healthcare services and activity support programs must be provided for these patients. Moreover, measures to improve individuals’ subjective health status must be considered.

Those with limited activity were more likely to have problems with mobility, self-care, usual activities, pain/discomfort, and anxiety/depression. Hence, interventions to assist or improve the limitations in activity of patients with multimorbidity must be implemented with the highest priority. Specific strategies, such as visiting healthcare or medical services, leisure activity assistance programs, or transportation assistance programs for multimorbidity patients with limited activity must be established. Access to healthcare must be improved to prevent unmet medical needs of those with limited activity.

For multimorbidity patients with multiple chronic diseases, some suggest that another level of treatment deviating from existing treatment methods must be provided, as they regard multimorbidity as a systematic disease where chronic diseases affect each other, rather than a combination of independent chronic diseases [35]. The results of this study are meaningful in this respect, as we targeted patients with multimorbidity according to the standards of the WHO; accordingly, in a study targeting the general population, patients with at least three chronic diseases are considered to have multimorbidity; however, we did not include certain chronic diseases such as chronic obstructive pulmonary disease. Studies drawn from a broader population of patients should thus be conducted in the future. Although we used data from a reliable and representative large-scale national dataset, not all the dimension for Andersen’s model were met. Furthermore, the design of our study was cross-sectional, which limits interpretations regarding causality.

## 5. Conclusions and Recommendation

Predisposing, enabling, and need factors along with health behaviors need to be examined when investigating the HRQoL of patients with multimorbidity, with a focus on the specific HRQoL dimensions in order to further provide personalized care. Individuals with limited activity were more likely to experience problems with mobility, self-care, usual activities, pain/discomfort, and anxiety/depression; hence, interventions to address the limited activity of these patients should be a top priority.

In Korea, the frequency of using healthcare services may increase through self-referral or cross-referral when treating multimorbidity due to the limitations of the primary care system [8]. Patients may still experience unmet medical needs despite excessive healthcare service use. Therefore, multimorbidity needs to be treated in primary care. In one pilot project conducted in Korea, the results showed that a multimorbidity support center model based on primary care could efficiently manage multimorbidity patients and reduce the use of unnecessary medical services by controlling the selection of appropriate targets, multidisciplinary management of healthcare fields, establishing a personalized care plan for each patient, and integrating treatment and patient education [35].

## Figures and Tables

**Table 1:** General characteristics of patients with multimorbidity (*N* = 328) (South Korea, 2014–2016).

Variable		Class	*n* (%)	Mean ± SD
Predisposing factors	Gender	Male	164 (50.0)	
		Female	164 (50.0)	
	Age	Under 65	88 (26.8)	
		65 years or older	240 (73.2)	
	Education level	Below high school graduate	284 (87.6)	
		Above high school graduate	40 (12.4)	
	Marital status	With spouse	225 (68.6)	
		No spouse	103 (31.4)	
Enabling factors	Basic living security	No	277 (84.5)	
		Yes	51 (15.5)	
	Health insurance	National Health Insurance	281 (87.8)	
		Medicaid	39 (12.2)	
	Private insurance	Yes	117 (35.7)	
		No	211 (64.3)	
	Household income	Low	161 (49.1)	210.1 ± 244.8 ^a^
		Mid-low	81 (24.7)	
		Mid-high	50 (15.2)	
		High	36 (11.0)	
	Living with family	Yes	257 (78.3)	
		No	71 (21.7)	
	Limited activity	No	239 (72.9)	
		Yes	89 (27.1)	
Need factors	Number of chronic diseases	3	275 (83.9)	3.2 ± 0.4
		4	46 (14.0)	
		5	7 (2.1)	
	Subjective health status	Good	123 (37.5)	
		Poor	205 (62.5)	
	Unmet medical needs	No	287 (87.5)	
		Yes	41 (12.5)	
Health behaviors	Smoking	No	262 (81.6)	
		Yes	59 (18.4)	
	Drinking	No	168 (52.3)	
		Yes	153 (47.7)	
	Physical activity	Yes	42 (12.9)	
		No	283 (87.1)	
	Health screenings	Yes	191 (58.4)	
		No	136 (41.6)	
EQ-5D-L3	Mobility	No problem	153 (46.8)	
		Problem	174 (53.2)	
	Self-care	No problem	256 (78.0)	
		Problem	72 (22.0)	
	Usual activity	No problem	187 (57.0)	
		Problem	141 (43.0)	
	Pain/discomfort	No problem	176 (53.7)	
		Problem	152 (46.3)	
	Anxiety/depression	No problem	240 (73.2)	
		Problem	88 (26.8)	

^a^ 10,000 South Korean won; SD: standard deviation.

**Table 2:** Factors affecting mobility dimension of health-related quality of life in patients with multimorbidity according to Andersen’s model (South Korea, 2014–2016).

Variable		Model 1	Model 2	Model 3	Model 4
		OR (*p*) (95% CI)	OR (*p*) (95% CI)	OR (*p*) (95% CI)	OR (*p*) (95% CI)
Predisposing factors	Gender (ref = Male)	1.06 (0.820) (0.65, 1.72)	1.12 (0.661) (0.67, 1.88)	1.04 (0.888) (0.61, 1.77)	1.21 (0.543) (0.66, 2.19)
	Age (ref = Under 65)	2.48 (0.001) (1.47, 4.18)	1.95 (0.026) (1.08, 3.51)	2.33 (0.007) (1.26, 4.31)	2.30 (0.011) (1.21, 4.37)
	Education level (ref = Below high school graduate)	0.28 (0.001) (0.13, 0.61)	0.31 (0.006) (0.14, 0.72)	0.36 (0.019) (0.15, 0.84)	0.43 (0.067) (0.18, 1.06)
	Marital status (ref = With spouse)	1.80 (0.028) (1.07, 3.04)	1.28 (0.503) (0.62, 2.62)	1.37 (0.407) (0.65, 2.88)	1.22 (0.621) (0.56, 2.66)
Enabling factors	Basic living security (ref = No)		0.87 (0.775) (0.32, 2.33)	0.77 (0.629) (0.27, 2.21)	0.77 (0.627) (0.27, 2.22)
	Health insurance (ref = National health insurance)		2.32 (0.142) (0.75, 7.17)	2.48 (0.135) (0.75, 8.17)	2.31 (0.171) (0.70, 7.68)
	Private insurance (ref = Yes)		1.85 (0.029) (1.07, 3.20)	1.83 (0.035) (1.04, 3.20)	1.85 (0.037) (1.04, 3.30)
	Household income (monthly average)		1.00(0.950) (1.00, 1.00)	1.00 (0.804) (1.00. 1.00)	1.00 (0.594) (1.00, 1.00)
	Living with family (ref = Yes)		1.37 (0.455) (0.60, 3.12)	1.44 (0.397) (0.62, 3.36)	1.59 (0.296) (0.67, 3.81)
	Limited activity (ref = No)		3.26 (<0.001) (1.82, 5.86)	2.31 (0.008) (1.24, 4.31)	2.43 (0.007) (1.27, 4.62)
Need factors	Number of chronic diseases			1.19 (0.569) (0.66, 2.12)	1.20 (0.552) (0.66, 2.19)
	Subjective health status (ref = Good)			2.00 (0.012) (1.17, 3.42)	1.78 (0.042) (1.02, 3.10)
	Unmet medical needs (ref = No)			2.47 (0.044) (1.03, 5.93)	2.78 (0.025) (1.14, 6.79)
Health behaviors	Smoking (ref = No)				1.95 (0.075) (0.94, 4.08)
	Drinking (ref = No)				0.80 (0.437) (0.46, 1.41)
	Physical activity (ref = Yes)				2.19 (0.073) (0.93, 5.16)
	Health screenings (ref = Yes)				1.36 (0.260) (0.80, 2.33)
Wald (*p*)		28.41 (<0.001)	47.31 (<0.001)	54.42 (<0.001)	57.51 (<0.001)
Nagelkerke’s R^2^		0.130	0.237	0.281	0.308

OR: odds ratio; CI: confidence interval.

**Table 3:** Factors affecting self-care dimension of health-related quality of life in patients with multimorbidity according to Andersen’s model (South Korea, 2014–2016).

Variable		Model 1	Model 2	Model 3	Model 4
		OR (*p*) (95% CI)	OR (*p*) (95% CI)	OR (*p*) (95% CI)	OR (*p*) (95% CI)
Predisposing factors	Gender (ref = Male)	0.78 (0.397) (0.44, 1.38)	0.88 (0.675) (0.47, 1.63)	0.83 (0.557) (0.44, 1.55)	0.99 (0.970) (0.48, 2.05)
	Age (ref = Under 65)	1.82 (0.079) (0.93, 3.53)	1.07 (0.862) (0.50, 2.30)	1.15 (0.722) (0.53, 2.54)	1.26 (0.583) (0.55, 2.85)
	Education level (ref = Below high school graduate)	0.50 (0.170) (0.19, 1.35)	0.77 (0.629) (0.26, 2.26)	0.78 (0.668) (0.25, 2.41)	0.70 (0.559) (0.22, 2.29)
	Marital status (ref = With spouse)	1.57 (0.134) (0.87, 2.82)	1.50 (0.375) (0.61, 3.64)	1.48 (0.392) (0.61, 3.61)	1.16 (0.757) (0.45, 3.02)
Enabling factors	Basic living security (ref = No)		0.56 (0.312) (0.18, 1.72)	0.54 (0.303) (0.17, 1.75)	0.52 (0.282) (0.15, 1.73)
	Health insurance (ref = National health insurance)		1.25 (0.708) (0.39, 3.97)	1.10 (0.875) (0.33, 3.71)	0.89 (0.849) (0.25, 3.14)
	Private insurance (ref = Yes)		2.58 (0.014) (1.12, 5.49)	2.69 (0.011) (1.25, 5.80)	2.76 (0.010) (1.27, 6.01)
	Household income (monthly average)		1.00 (0.099) (1.00, 1.00)	1.00 (0.172) (1.00, 1.00)	1.00 (0.234) (1.00, 1.00)
	Living with family (ref = Yes)		0.82 (0.700) (0.31, 2.20)	0.93 (0.889) (0.35, 2.50)	1.20 (0.725) (0.43, 3.32)
	Limited activity (ref = No)		4.21 (<0.001) (2.33, 7.60)	3.59 (<0.001) (1.91, 6.73)	4.15 (<0.001) (2.15, 8.01)
Need factors	Number of chronic diseases			0.65 (0.229) (0.32, 1.32)	0.65 (0.234) (0.32, 1.33)
	Subjective health status (ref = Good)			1.96 (0.059) (0.98, 3.92)	1.78 (0.112) (0.87, 3.64)
	Unmet medical needs (ref = No)			1.92 (0.130) (0.83, 4.46)	2.12 (0.094) (0.88, 5.08)
Health behaviors	Smoking (ref = No)				1.73 (0.201) (0.75, 4.02)
	Drinking (ref = No)				0.94 (0.869) (0.46, 1.92)
	Physical activity (ref = Yes)				0.69 (0.449) (0.26, 1.82)
	Health screenings (ref = Yes)				1.50 (0.206) (0.80, 2.81)
Wald (p)		7.59 (0.108)	37.66 (<0.001)	41.83 (<0.001)	44.87 (<0.001)
Nagelkerke’s R^2^		0.039	0.213	0.245	0.272

OR: odds ratio; CI: confidence interval.

**Table 4:** Factors affecting usual activity dimension of health-related quality of life in patients with multimorbidity according to Andersen’s model (South Korea, 2014–2016).

Variable		Model 1	Model 2	Model 3	Model 4
		OR (*p*) (95% CI)	OR (*p*) (95% CI)	OR (*p*) (95% CI)	OR (*p*) (95% CI)
Predisposing factors	Gender (ref = Male)	1.01 (0.973) (0.63, 1.62)	1.15 (0.590) (0.69, 1.93)	1.03 (0.921) (0.60, 1.76)	1.29 (0.425) (0.69, 2.39)
	Age (ref = Under 65)	1.47 (0.144) (0.88, 2.45)	1.09 (0.772) (0.60, 1.99)	1.26 (0.474) (0.67, 2.34)	1.40 (0.313) (0.73, 2.71)
	Education level (ref = Below high school graduate)	0.51 (0.074) (0.24, 1.07)	0.67 (0.328) (0.29, 1.51)	0.80 (0.609) (0.34, 1.88)	0.77 (0.581) (0.31, 1.92)
	Marital status (ref = With spouse)	1.46 (0.136) (0.89, 2.41)	1.43 (0.346) (0.68, 2.97)	1.49 (0.306) (0.70, 3.19)	1.31 (0.513) (0.58, 2.94)
Enabling factors	Basic living security (ref = No)		0.37 (0.065) (0.13, 1.07)	0.31 (0.038) (0.10, 0.94)	0.30 (0.037) (0.10, 0.93)
	Health insurance (ref = National health insurance)		3.13 (0.050) (1.00, 9.82)	3.75 (0.033) (1.12, 12.60)	3.15 (0.063) (0.94, 10.56)
	Private insurance (ref = Yes)		1.81 (0.040) (1.03, 3.19)	1.77 (0.056) (0.99, 3.19)	1.83 (0.054) (0.99, 3.37)
	Household income (monthly average)		1.00 (0.525) (1.00, 1.00)	1.00 (0.628) (1.00, 1.00)	1.00 (0.943) (1.00, 1.00)
	Living with family (ref = Yes)		0.84 (0.688) (0.37, 1.94)	0.89 (0.798) (0.38, 2.11)	1.05 (0.921) (0.43, 2.54)
	Limited activity (ref = No)		5.05 (<0.001) (2.87, 8.87)	3.69 (<0.001) (2.03, 6.69)	4.22 (<0.001) (2.26, 7.89)
Need factors	Number of chronic diseases			1.49 (0.186) (0.83, 2.67)	1.55 (0.159) (0.84, 2.86)
	Subjective health status (ref = Good)			3.05 (<0.001) (1.75, 5.34)	2.81 (<0.001) (1.58, 5.00)
	Unmet medical needs (ref = No)			1.13 (0.763) (0.50, 2.57)	1.26 (0.588) (0.54, 2.94)
Health behaviors	Smoking (ref = No)				2.56 (0.013) (1.22, 5.38)
	Drinking (ref = No)				0.91 (0.757) (0.50, 1.65)
	Physical activity (ref = Yes)				0.85 (0.700) (0.36, 1.98)
	Health screenings (ref = Yes)				1.69 (0.061) (0.98, 2.93)
Wald (p)		8.83 (0.066)	46.88 (<0.001)	57.93 (<0.001)	63.39 (<0.001)
Nagelkerke’s R^2^		0.038	0.225	0.291	0.331

OR: odds ratio; CI: confidence interval.

**Table 5:** Factors affecting pain/discomfort dimension of health-related quality of life in patients with multimorbidity according to Andersen’s model (South Korea, 2014–2016).

Variable		Model 1	Model 2	Model 3	Model 4
		OR (*p*) (95% CI)	OR (*p*) (95% CI)	OR (*p*) (95% CI)	OR (*p*) (95% CI)
Predisposing factors	Gender (ref = Male)	1.76 (0.018) (1.10, 2.82)	2.14 (0.003) (1.28, 3.56)	2.04 (0.008) (1.21, 3.43)	2.14 (0.012) (1.19, 3.87)
	Age (ref = Under 65)	1.25 (0.398) (0.75, 2.07)	1.37 (0.296) (0.76, 2.48)	1.59 (0.138) (0.86, 2.93)	1.68 (0.108) (0.89, 3.18)
	Education level (ref = Below high school graduate)	0.56 (0.120) (0.27, 1.16)	0.54 (0.142) (0.24, 1.23)	0.64 (0.284) (0.28, 1.46)	0.57 (0.212) (0.24, 1.38)
	Marital status (ref = With spouse)	1.43 (0.158) (0.87, 2.37)	1.14 (0.716) (0.56, 2.33)	1.23 (0.579) (0.59, 2.56)	1.20 (0.636) (0.56, 2.56)
Enabling factors	Basic living security (ref = No)		0.51 (0.185) (0.18, 1.39)	0.42 (0.109) (0.15, 1.21)	0.41 (0.099) (0.14, 1.18)
	Health insurance (ref = National health insurance)		5.14 (0.005) (1.63, 16.25)	5.87 (0.004) (1.76, 19.58)	5.31 (0.007) (1.58, 17.88)
	Private insurance (ref = Yes)		0.90 (0.709) (0.51, 1.58)	0.86 (0.593) (0.48, 1.52)	0.83 (0.536) (0.46, 1.49)
	Household income (monthly average)		1.00 (0.150) (1.00, 1.00)	1.00 (0.102) (1.00, 1.00)	1.00 (0.074) (1.00, 1.00)
	Living with family (ref = Yes)		1.40 (0.419) (0.62, 3.15)	1.46 (0.373) (0.34, 3.32)	1.61 (0.267) (0.69, 3.75)
	Limited activity (ref = No)		4.44 (<0.001) (2.50, 7.89)	3.29 (<0.001) (1.80, 6.02)	3.64 (<0.001) (1.94, 6.81)
Need factors	Number of chronic diseases			1.27 (0.423) (0.71, 2.28)	1.29 (0.402) (0.71, 2.36)
	Subjective health status (ref = Good)			2.17 (0.005) (1.26, 3.72)	2.05 (0.011) (1.18, 3.58)
	Unmet medical needs (ref = No)			1.96 (0.106) (0.87, 4.44)	2.08 (0.086) (0.90, 4.79)
Health behaviors	Smoking (ref = No)				1.48 (0.290) (0.72, 3.05)
	Drinking (ref = No)				0.81 (0.464) (0.46, 1.42)
	Physical activity (ref = Yes)				0.64 (0.279) (0.28, 1.44)
	Health screenings (ref = Yes)				1.13 (0.661) (0.66, 1.92)
Wald (p)		15.36 (0.004)	45.96 (<0.001)	53.00 (<0.001)	55.16 (<0.001)
Nagelkerke’s R^2^		0.066	0.227	0.272	0.292

OR: odds ratio; CI: confidence interval.

**Table 6:** Factors affecting anxiety/depression dimension of health-related quality of life in patients with multimorbidity according to Andersen’s model (South Korea, 2014–2016).

Variable		Model 1	Model 2	Model 3	Model 4
		OR (*p*) (95% CI)	OR (*p*) (95% CI)	OR (*p*) (95% CI)	OR (*p*) (95% CI)
Predisposing factors	Gender (ref = Male)	1.18 (0.536) (0.70, 2.00)	1.20 (0.514) (0.69, 2.08)	1.10 (0.754) (0.62, 1.94)	1.27 (0.469) (0.66, 2.43)
	Age (ref = Under 65)	0.60 (0.062) (0.35, 1.03)	0.54 (0.048) (0.29, 1.00)	0.62 (0.141) (0.33, 1.17)	0.67 (0.229) (0.35, 1.29)
	Education level (ref = Below high school graduate)	0.71 (0.413) (0.31, 1.63)	0.86 (0.742) (0.36, 2.08)	1.03 (0.957) (0.41, 2.53)	0.94 (0.892) (0.37, 2.41)
	Marital status (ref = With spouse)	1.26 (0.417) (0.73, 2.18)	1.09 (0.833) (0.50, 2.37)	1.17 (0.702) (0.52, 2.63)	1.20 (0.664) (0.52, 2.78)
Enabling factors	Basic living security (ref = No)		0.63 (0.386) (0.22, 1.80)	0.51 (0.239) (0.17, 1.56)	0.53 (0.269) (0.17, 1.63)
	Health insurance (ref = national health insurance)		2.18 (0.164) (0.73, 6.52)	2.18 (0.184) (0.69, 6.90)	2.16 (0.202) (0.66, 7.03)
	Private insurance (ref = Yes)		0.85 (0.607) (0.47, 1.56)	0.82 (0.524) (0.44, 1.51)	0.83 (0.549) (0.45, 1.54)
	Household income (monthly average)		1.00 (0.509) (1.00, 1.00)	1.00 (0.778) (1.00, 1.00)	1.00 (0.804) (1.00, 1.00)
	Living with family (ref = Yes)		0.92 (0.860) (0.38, 2.23)	1.04 (0.936) (0.42, 2.55)	1.01 (0.985) (0.40, 2.53)
	Limited activity (ref = No)		2.74 (<0.001) (1.57, 4.79)	1.91 (0.037) (1.04, 3.50)	2.04 (0.024) (1.10, 3.80)
Need factors	Number of chronic diseases			1.07 (0.826) (0.58, 1.97)	1.05 (0.875) (0.57, 1.95)
	Subjective health status (ref = Good)			2.33 (0.009) (1.23, 4.39)	2.26 (0.013) (1.19, 4.32)
	Unmet medical needs (ref = No)			2.91 (0.006) (1.35, 6.29)	2.95 (0.007) (1.35, 6.47)
Health behaviors	Smoking (ref = No)				1.54 (0.258) (0.73, 3.23)
	Drinking (ref = No)				1.06 (0.863) (0.57, 1.97)
	Physical activity (ref = Yes)				0.72 (0.434) (0.32, 1.64)
	Health screenings (ref = Yes)				0.95 (0.867) (0.54, 1.68)
Wald (p)		5.49 (0.241)	19.96 (0.030)	31.82 (0.003)	33.48 (0.010)
Nagelkerke’s R^2^		0.025	0.095	0.165	0.178

OR: odds ratio; CI: confidence interval.

## Data Availability

Data were obtained from KCDC and are available from https://knhanes.cdc.go.kr/knhanes/sub03/sub03_02_05.do (accessed on 5 September 2018).
